# The Role of Women’s Empowerment in Fertility Preferences and Outcomes: Analysis of the 2017 Indonesia Demographic and Health Survey

**DOI:** 10.1186/s12905-025-03748-6

**Published:** 2025-04-30

**Authors:** Sitta Fiakhsani Taqwim, Masoud Vaezghasemi, Sara Castel-Feced, Fatwa Sari Tetra Dewi, Julia Schröders

**Affiliations:** 1 Department of Epidemiology and Global Health, Umeå University, Umeå, 90 187 Sweden; 2 Department of Statistical Methods, University of Zaragoza, Zaragoza, 50005 Spain; 3 Department of Health Behavior, Environment and Social Medicine, Faculty of Medicine, Public Health and Nursing, Universitas Gadjah Mada, Yogyakarta, Indonesia

**Keywords:** Women’s empowerment, Fertility preference, Reproductive autonomy, Gender-based violence, Decision-making, Indonesia, Demographic and Health Survey

## Abstract

**Background:**

With a population of 275 million, Indonesia is the world’s fourth most populous country and has made considerable efforts to reduce its Total Fertility Rate from 5.6 in 1971 to a target of 2.1 by 2024. Women’s empowerment has been identified as a critical factor influencing fertility dynamics, gender equality, reproductive autonomy, and broader socioeconomic development. This study examines the association between four dimensions of women’s empowerment and three fertility-related outcomes among married women aged 22 years and older in Indonesia.

**Methods:**

We used cross-sectional data from 34,017 married women participating in the 2017 Indonesia Demographic and Health Survey (IDHS). An outcome-wide analytical approach was adopted to explore three outcomes: total number of children ever born, ideal number of children, and fertility preference alignment, i.e. whether actual fertility matched stated preferences. Four empowerment domains were assessed: household decision-making, attitudes toward wife beating, attitudes toward refusing sex, and labour force participation. Stepwise multivariate Poisson regression modelling was applied, adjusting for key demographic and socioeconomic covariates.

**Results:**

Our study found that the association between each type of women’s empowerment and fertility-related outcomes varied, reinforcing the notion that empowerment does not uniformly affect reproductive behaviour. Among the four empowerment indicators, rejecting all justifications for wife beating emerged as the most consistent and significant predictor across all fertility outcomes. It was association with fewer children ever born (β = 0.03), a lower ideal number of children (β = 0.04), and a higher likelihood of meeting fertility preferences (PR = 1.02). Attitudes toward refusing sex were also significantly associated with fewer children (β = 0.02) and lower fertility ideals (β = 0.07). However, participation in decision-making and labour force participation showed mixed or non-significant associations, indicating that different empowerment dimensions may influence reproductive behaviour in diverse ways.

**Conclusions:**

Women’s empowerment - particularly in the domains of gender-based violence and sexual autonomy - is closely linked to fertility preferences and behaviours. The findings underscore that empowerment is a multidimensional construct, with varying influences across its domains. Strengthening women’s autonomy and addressing gender-based violence are essential steps toward enhancing reproductive rights and achieving Sustainable Development Goal 5 in Indonesia. Gender-sensitive data systems and interventions tailored to different aspects of empowerment are urgently needed.

**Supplementary Information:**

The online version contains supplementary material available at 10.1186/s12905-025-03748-6.

## Introduction

The two-child policy in Indonesia was introduced in the late 1970s with a slogan “small, happy, and prosperous family”. The Indonesian Population Census in 1971 showed that a large family size contributed a heavy burden for individual households and national economy [1]. Indonesia has successfully decreased the Total Fertility Rate (TFR) from 5.61 in 1971 to 2.18 in 2020 [2]. A previous study by Permana & Westoff [3] reported that women in Java and Bali tend to desire less children than other regions and 40% of married women viewed having two children as ideal.

The control of fertility can facilitate greater access for women to educational, economic, and civic opportunities, particularly as gender roles and expectations evolve. In many settings, including Indonesia, traditional views that primarily assign women reproductive and caregiving responsibilities are shifting. This study draws on Malhotra’s framework [4], which outlines how changing values around childbearing, increased autonomy in reproductive decisions, and access to contraception can transform gender systems and expand women’s agency. The increasing availability and use of contraception was the important determinant of fertility decline in developing world [5]. The use of modern contraceptive in Indonesia in 1973 was only 7% and increased into 54% in 2018 and recent study revealed that the contraceptive use in Indonesia was 64% [6].

Women’s empowerment is a key element in advancing human development and reducing poverty [7]. It is best understood as an ongoing process rather than a fixed state, involving a fundamental shift for individuals who have previously been denied the ability to make strategic life choices. Such choices include the freedom of movement, the decision to have children, and how many children to have—each with significant consequences for women’s autonomy and well-being [8–10]. In this study, empowerment is proxied through four indicators: participation in decision-making, attitudes toward wife beating and refusing sex, and labour force participation. Although empowerment is inherently complex and context-dependent, these proxies align with those used in similar research across diverse settings [7, 11, 15, 18–20].

High fertility rates, meanwhile, are often influenced by sociocultural and economic factors. In many settings, having multiple children–especially sons– continues to be seen as a means to secure a woman’s status within her family or community [11–14]. In some cultures, sons are preferred for economic, social, and even spiritual reasons, perpetuating gender inequality across generations. Additionally, children are often perceived as contributors to household labour or future income, especially in lower-income settings, further reinforcing the desire for large families [15]. Previous studies have proposed that increased women’s empowerment is linked to declining fertility, highlighting the importance of understanding this relationship in the Indonesian context [16–18].

Women who have greater decision-making power in the domestic aspects were reported to have low levels of fertility [16]. The women’s acceptance of domestic violence was hypothesized to reflect the women’s perspective if she was being abused in different circumstances. The culture of silence due to prevalent societal norm may prevent women from defending themselves in undesirable situations [21]. A recent study found evidence of a direct causal link between family size and intimate partner violence [22]. Another study in Colombia [23] reported that 55% of women had at least one unintended pregnancy and 38% had been experiencing physical and sexual abuse from their partner. The female attitude towards refusing sex with their spouse may indicate their awareness of sexual health and reproductive rights. Several studies have reported that women’s ability to make their own informed decision related to marital sexual relations was associated with fertility behaviours [24–27]. Several studies in Egypt, Ghana and Bangladesh showed that formal employment had the most consistent empowering implications [28]. While a study in six Pacific Island Countries reported that 1% increase in female labour force participation decreased fertility on average 0.014% [29].

In the past decade many research or pooled studies about empowerment and reproductive health have focused on the African and South Asia settings as high fertility countries [7, 14, 15, 18, 24, 26, 30–41]. Comprehensive studies relating to women’s empowerment and fertility behaviours in South-East Asia, particularly in the Indonesian context are still limited [12, 42–45] It remains unclear how women’s empowerment indicators are associated with fertility preferences due to the complexity of its dimension and potential overlapping associations.

Indonesia as an upper-middle income country seems out of target for this focus of research despite the fact that the country’s achievement of Sustainable Development Goals (SDGs) number 5 is still far behind [46]. The important goals that need to be improved by policymakers in the country are eliminating all forms of violence against all women and girls and ensuring universal access to reproductive rights. There is no indicator reported related to domestic violence, while the ratio of female-to-male labour force participation rate was 65.4% in 2022 and considered as moderately improving from 55% in 2011 [46, 47]. The indicators of women’s empowerment and the fertility outcomes and preferences to be measured in this study was in line with the SDGs goal number 5. Additionally, reproductive empowerment defined as women’s capacity to make informed decision about their reproductive lives should be perceived as both a process and an outcome and involving a right-based approach [48]. The aim of this study was to examine the association between four women’s empowerment indicators (participation in decision-making, attitude towards wife beating, attitude towards refusing sex and labour force participation) and three fertility outcomes and preferences (total number of children ever born, ideal number of children and fertility preference) among married women aged ≥ 22 years old in Indonesia.

## Methods

### Study design, sampling, study population, and data collection

**Fig. 1:**
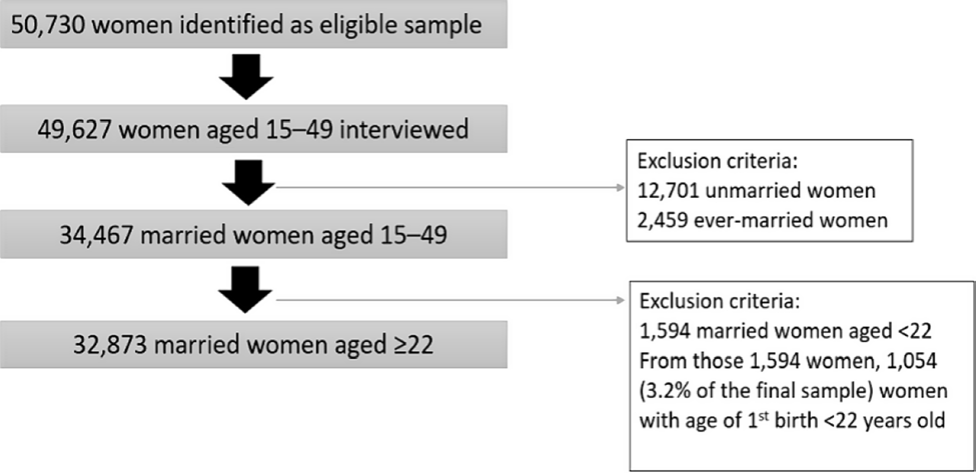
Flowchart of study respondents and exclusion criteria

This cross-sectional study utilized data from the 2017 Indonesia Demographic and Health Survey (IDHS). The data was accessed through permission from the DHS website (https://dhsprogram.com/). The DHS program has representative data on population, health, and nutrition from more than 400 surveys in over 90 countries, including Indonesia. The sampling design of the 2017 IDHS was representative of the population aged 15–49 at the national level, provincial level and for urban and rural areas. The sample represented 34 provinces in the country. The DHS survey applied weighting in order to achieve a more accurate representative proportion of the sample from different provinces for reliable statistics. For this study, before conducting the analysis, the data was cleaned up and narrowed to acquire an appropriate sample (Fig. 1). The population of interest was married women aged ≥ 22 years old. The justification for selecting 22 years and older was based on the median age at first birth which is 22.4 years according to the IDHS 2017 report [49] and the data-driven mean age of the first birth of 21.6 years (SD 4.36) in our sample.

### Outcome variables

**Fig. 2:**
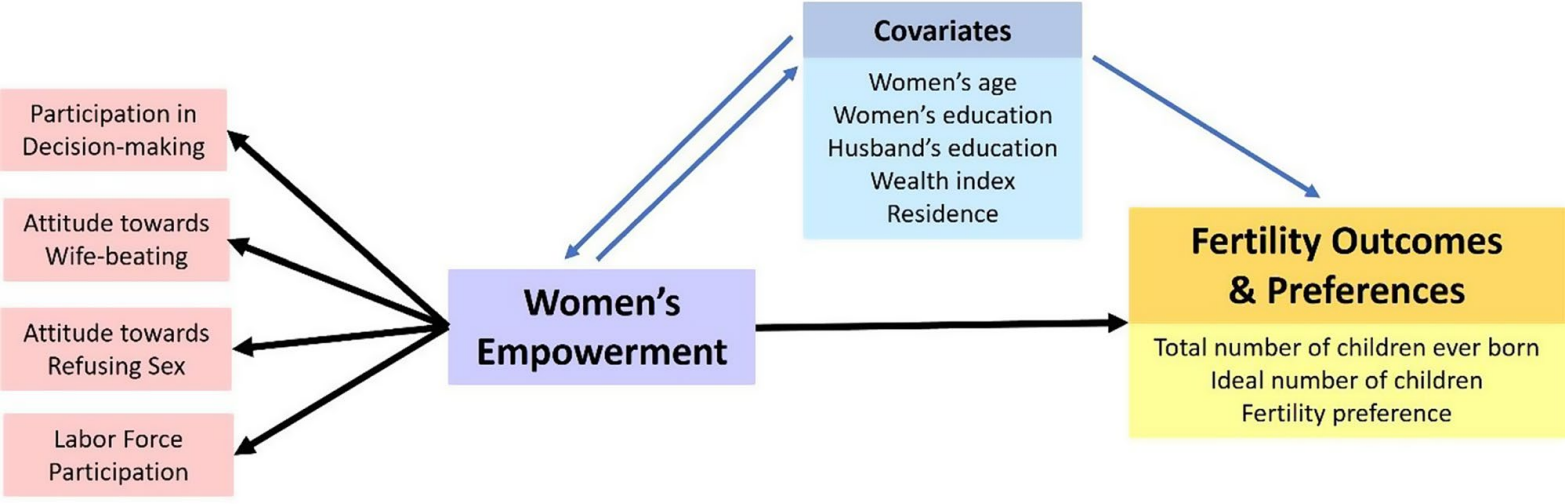
Analytical framework of women’s empowerment factors and fertility outcomes and preferences

This study applied an outcome-wide analytical approach [50] focusing on three fertility-related outcomes: the total number of children ever born, women’s perception of the ideal number of children, and alignment between actual and preferred fertility—referred to here as fertility preference (Fig. 2).

The first two outcomes—total number of children ever born and the perceived ideal number of children—were treated as count data, based on direct numeric responses from the 2017 IDHS. The third outcome, fertility preference, was constructed by calculating the difference between the total number of children ever born and the ideal number of children reported by each woman. If the number of children ever born matched the ideal number, the respondent was classified as having met their fertility preference (coded as 0, reference category). All other responses, where the reported number of children differed from the ideal, were considered as not meeting their fertility preference (coded as 1).

This approach allowed us to explore not only fertility behaviour but also the extent to which women’s reproductive outcomes aligned with their stated preferences.

### Independent variables

This study used four proxy indicators to measure women’s empowerment: participation in household decision-making, attitude toward wife beating, attitude toward refusing sex, and labour force participation. These indicators were selected based on their frequent use in previous studies and their availability in the 2017 Indonesia Demographic and Health Survey (IDHS). While we did not apply a single established empowerment framework, our choice was informed by global literature and surveys—particularly DHS analyses—that consistently utilize these dimensions to capture core aspects of empowerment. A conceptual framework (Fig. 2) was developed to guide the analytical approach and visualize the hypothesized relationships between empowerment dimensions and fertility outcomes. Each of four women’s empowerment indicator has different number of sub-indicators as showed in Table 1.

**Table 1:** Women’s empowerment indicator in the Woman’s questionnaire 2017 IDHS

No.	Women’s Empowerment Indicators	Response	
		No	Yes
*Decision-making participation*			
1.	Decision-making alone or jointly in women’s own health care	1	0
2.	Decision-making alone or jointly in large household purchases	1	0
3.	Decision-making alone or jointly in visits to family or friends	1	0
*Attitude towards wife-beating*			
4.	Agree that a husband is justified in hitting/beating his wife if she goes out without telling him	0	1
5.	Agree that a husband is justified in hitting/beating his wife if she neglects the children	0	1
6.	Agree that a husband is justified in hitting/beating his wife if she argues with him	0	1
7.	Agree that a husband is justified in hitting/beating his wife if she refuses to have sex with him	0	1
8.	Agree that a husband is justified in hitting/beating his wife if she burns the food	0	1
*Attitude towards refusing sex with husband*			
9.	Wife is justified in refusing to have sex with her husband when she knows her husband has a sexually transmitted disease	1	0
10.	Wife is justified in refusing to have sex with her husband when she knows her husband has sex with other women	1	0
*Labor force participation*			
11.	Worked in the last 12 months	1	0

Note:

The reference coded as 0 when women considered as empowered

For decision-making participation, if women had no power were coded as 1

For attitude towards wife beating, if women agree then it is considered as disempowered and coded as 1

For attitude towards refusing sex with husband, if women disagree regarding refusing sex with husband it is considered as disempowered and coded as 1

For labour force participation, if women did not work then coded as 1

### Covariates

The covariates were selected to represent the respondent’s general demographic and socioeconomic background. The covariates used in this study were age groups, wife’s education, husband’s education, wealth index (quintiles) and residence.

### Statistical analysis

The analysis used sampling weights to adjust the IDHS data in order to get statistics which were representative and resembled the true distribution in Indonesia. All the selected variables representing the characteristics of the women as respondents were presented in frequencies, percentage and mean (SD) value.

As a part of the descriptive statistics, a women’s empowerment index was created. The label or code of eleven independent variables as in Table 1 was reversed with women who were categorized as empowered given score 1 for each question and the non-empowered group coded as 0. The women who had score 11 were perceived as the most empowered group in this study population.

The analytical approach involved three models for each outcome variable. Univariable regression was first performed for each independent variable and covariate. Multivariable regression analyses were then conducted using three models: Model 1 included only the women’s empowerment variables; Model 2 included all covariates; and Model 3 was the full model, combining empowerment variables and covariates.

Given the nature of the outcome variables, we used different regression approaches. Poisson regression was applied for two outcomes—total number of children ever born and ideal number of children—reporting beta coefficients. For the binary outcome, fertility preference, we estimated prevalence ratios (PRs) using a generalized linear model (GLM) with a Poisson distribution, log link function, and robust standard errors, a method also known as modified Poisson regression. This approach is preferred over logistic regression when the outcome is not rare, as odds ratios may overestimate the association in cross-sectional studies [51]. All analyses and data visualizations were performed using R version 4.3.3. Statistical significance was defined as *p* < 0.05.

## Results

### Descriptive statistics

The majority of the women in the sample population in this study were considered as empowered. For instance, more than 60% of the women in this study participated in decision-making, disagreeing with any reasons of wife beating, and had agreement that women were justified to refuse sexual relation with their husband for particular reasons. The labour force participation measurement showed that approximately 57% women in this study had employment. The sociodemographic factors showed that the mean of total number of children ever born and ideal number of children were lower in the youngest group, women and their husband who had higher education, the richest and those who lived in the urban area compared to the counterparts as showed in the Table 2.

**Table 2:** Descriptive statistics (Weighted 2017 IDHS Data)

Variables	Labels	Total *N* (%)	Total Number of Children Ever Born	Ideal Number of Children	Fertility Preference	
			Mean (SD)	Mean (SD)	Met *n* (%)	Unmet *n* (%)
*Decision-making participation*	Yes No	23,312 (69) 10,652 (31)	2.25 (1.38) 2.28 (1.44)	2.98 (1.51) 3.05(1.51)	7,727 (33) 3,260 (31)	15,585 (67) 7,392 (69)
*Disagreeing with wife beating*	Yes No	23,311 (69) 10,690 (31)	2.24 (1.38) 2.30 (1.45)	2.95 (1.49) 3.10 (1.55)	7,808 (34) 3,184 (30)	15,503 (66) 7,506 (70)
*Attitude towards refusing sex*	Yes No	22,848 (67) 11,159 (33)	2.16 (1.30) 2.46 (1.56)	2.88 (1.40) 3.23 (1.69)	7,406 (32) 3,593 (32)	15,442 (68) 7,565 (68)
*Labor force participation*	Yes No	19,404 (57) 14,609 (43)	2.29 (1.43) 2.22 (1.36)	2.99 (1.50) 3.00 (1.52)	7,430 (33) 4,569 (31)	12,974 (67) 10,044 (69)
*Age groups*	22–29 30–39 40–49	9,092 (27) 13,933 (41) 10,993 (32)	1.35 (0.84) 2.33 (1.18) 2.93 (1.61)	2.81 (1.29) 2.98 (1.49) 3.19 (1.68)	1,646 (18) 5,106 (37) 4,247 (39)	7,446 (82) 8,827 (63) 6,745 (61)
*Education wife*	Higher Secondary Primary No Education	4,332 (13) 17,312 (51) 11,731 (34) 642 (2)	1.73 (1.13) 2.08 (1.24) 2.65 (1.52) 3.40 (2.15)	2.89 (1.29) 2.86 (1.41) 3.21 (1.66) 3.87 (1.91)	1,147 (26) 5,528 (32) 4,121 (35) 203 (32)	3,185 (74) 11,784 (68) 7,610 (65) 439 (68)
*Education husband*	Higher Secondary Primary No Education	4,180 (12) 17,697 (52) 11,487 (34) 599 (2)	1.93 (1.22) 2.12 (1.28) 2.55 (1.52) 3.16 (2.02)	3.00 (1.41) 2.88 (1.43) 3.15 (1.63) 3.52 (1.82)	1,156 (28) 5,653 (32) 3,966 (35) 199 (33)	3,023 (72) 12,043 (68) 7,521 (65) 401 (67)
*Wealth quintile*	Richest Richer Middle Poorer Poorest	7,217 (21) 7,311 (21) 7,051 (21) 6,647 (20) 5,792 (17)	2.10 (1.18) 2.10 (1.21) 2.16 (1.29) 2.31 (1.43) 2.71 (1.81)	2.84 (1.35) 2.87 (1.41) 2.96 (1.50) 3.01 (1.55) 3.39 (1.71)	2,503 (35) 2,435 (33) 2,216 (31) 2,160 (33) 1,684 (29)	4,713 (65) 4,876 (67) 4,835 (69) 4,486 (67) 4,107 (71)
*Place of residence*	Urban Rural	16,685 (49) 17,332 (51)	2.17 (1.30) 2.35 (1.48)	2.90 (1.44) 3.10 (1.57)	5,408 (32) 5,591 (32)	11,277 (68) 11,741 (68)
*Total*		*N* = 34,017 (100)	2.32 (1.40)	3.06 (1.52)	*N* = 10,999 (32)	*N* = 23,018 (68)

**Fig. 3:**
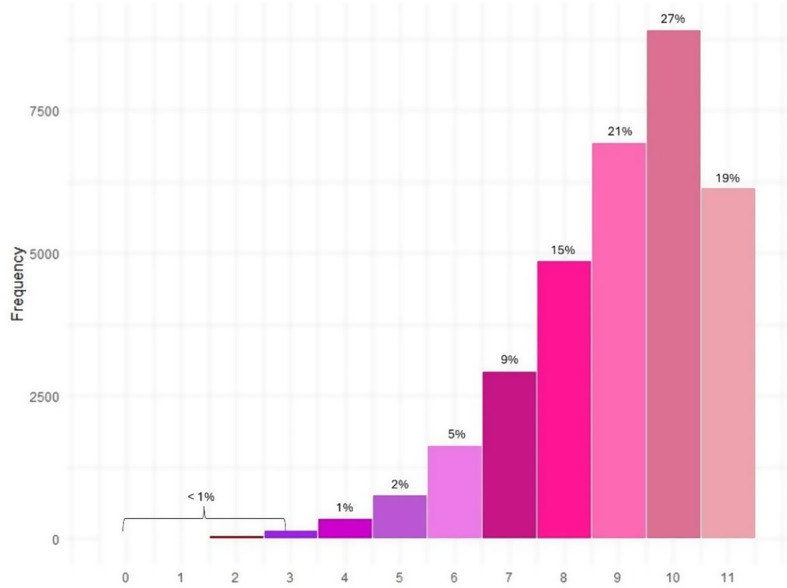
Women’s empowerment index

The majority of women in this sample population (27%) didnot reach the maximum level of empowerment with a score of 10 out of 11. Only 19% women in this study population had full score in all 11 questions (Fig. 3). The distribution of women with the highest empowerment level (score 11) were dominated by the age group 30–39, the richest, the secondary education level for both the wife and the husband, and living in urban areas.

### Analytical statistics

The women who disagreed with reasons justifying wife beating and had capacity to make own informed decisions regarding sexual relations with the husband or partner were less likely to have more children than women who were disempowered. Age and women’s education were other variables that were highly significant and contributed to the outcome number of children (Table 3). The older women and women with a lower level of education were more likely to have more children compared to the younger women and women with a higher education level.

The women’s empowerment indicator of disagreeing with reasons justifying wife beating (β = 0.04, 95% CI 0.03, 0.05) and making own informed decisions regarding sexual relations with husband (β = 0.07, 95% CI 0.06, 0.09) seemed to significantly and positively associate with the women’s perception regarding ideal number of children. The women who disagreed with all circumstances when their husband was justified for beating were less likely to desire more children compared to the group who only partly disagreed and totally agreed. The empowered group regarding attitude towards refusing sex with their husband had a lower ideal number of children than the women who were less empowered. Age was a statistically significant factor for desiring more children in the older group (β = 0.06, 95% CI 0.04, 0.07 for age 30–39 and β = 0.11, 95% CI 0.09, 0.13 for age 40–49) compared to the younger group as the reference.

**Fig. 4:**
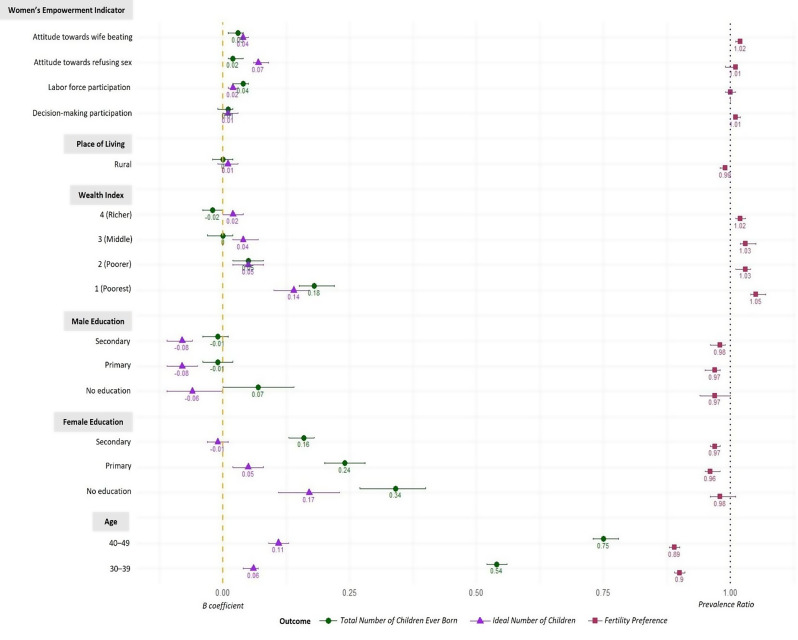
Forest Plot for Outcome Total Number of Children Ever Born, Ideal Number of Children, and Fertility Preference with 95% CI. Note: The reference used were the empowered group (for Women’s Empowerment indicator), urban area (for the place of living), 5 or richest (for the wealth index), higher education (for both male and female education), and 22–29 or the youngest (for the age group)

The fertility preference as the third outcome was measured by the prevalence ratio. Participation in decision-making (PR = 1.01, 95% CI 1.01, 1.02) and attitude towards wife beating (PR = 1.02, 95% CI 1.01, 1.02) were two statistically significant women’s empowerment indicators in all models and associated with the female’s fertility desire (Fig. 4). Women who agreed with any reasons for wife-beating were more likely to report did not achieve their fertility preference than women who disagreed in all circumstances that justified their husband in domestic violence. The results showed that an attitude of refusing sex with husband and labour force participation were not associated with the ability of women to achieve their fertility preference. Sociodemographic variables used as control measurement, i.e., women’s and husband’s education, wealth status in the household, and place of residence were not statistically significant in women’s fertility preference.

**Table 3:** Outcome for total number of children ever born, ideal number of children and fertility preference

Variables	Labels	Outcome Total Number of Children Ever Born Β coeff (95% CI)	Outcome Ideal Number of Children Β coeff (95% CI)	Outcome Fertility Preference PR (95% CI)
*Decision-making participation*	Yes No	– 0.01 (-0.01, 0.02)	– 0.01 (-0.00, 0.03)	– 1.01 (1.01, 1.02)
*Disagreeing with wife beating*	Yes No	– 0.03 (0.01, 0.04)	– 0.04 (0.03, 0.05)	– 1.02 (1.01, 1.02)
*Attitude towards refusing sex*	Yes No	– 0.02 (0.01, 0.04)	– 0.07 (0.06, 0.09)	– 1.01 (0.99, 1.01)
*Labor force participation*	Yes No	– 0.04 (0.02, 0.05)	– 0.02 (0.01, 0.03)	– 1.00 (0.99, 1.01)
*Age groups*	22–29 30–39 40–49	– 0.54 (0.52, 0.56) 0.75 (0.73, 0.78)	– 0.06 (0.04, 0.07) 0.11 (0.09, 0.13)	– 0.90 (0.89, 0.91) 0.89 (0.88, 0.90)
*Education wife*	Higher Secondary Primary No Education	– 0.16 (0.13, 0.18) 0.24 (0.20, 0.28) 0.34 (0.27, 0.40)	– -0.01 (-0.03, 0.01) 0.05 (0.02, 0.08) 0.17 (0.11, 0.23)	– 0.97 (0.96, 0.98) 0.96 (0.95, 0.98) 0.98 (0.96, 1.01)
*Education husband*	Higher Secondary Primary No Education	– -0.01 (-0.04, 0.01) -0.01 (-0.04, 0.02) 0.07 (0.00, 0.14)	– -0.08 (-0.11, -0.06) -0.08 (-0.11, -0.05) -0.06 (-0.11, 0.00)	– 0.98 (0.96, 0.99) 0.97 (0.95, 0.98) 0.97 (0.94, 1.00)
*Wealth quintile*	Richest Richer Middle Poorer Poorest	– -0.02 (-0.04, 0.00) 0.00 (-0.03, 0.02) 0.05 (0.02, 0.08) 0.18 (0.15, 0.22)	– 0.02 (-0.00, 0.04) 0.04 (0.02, 0.07) 0.05 (0.02, 0.08) 0.14 (0.10, 0.17)	– 1.02 (1.01, 1.03) 1.03 (1.02, 1.05) 1.03 (1.01, 1.04) 1.05 (1.04, 1.07)
*Place of residence*	Urban Rural	– 0.00 (-0.02, 0.02)	– 0.01 (-0.01, 0.03)	– 0.99 (0.98, 0.99)

## Discussion

### Main findings

This study found that the association between each type of women’s empowerment and fertility-related outcomes varied, reinforcing the notion that empowerment does not uniformly affect reproductive behaviour. Among the four women’s empowerment indicators, attitude towards wife beating and refusal of sex were significantly associated with at least one fertility-related outcome. However, participation in decision-making and labour force participation showed mixed or non-significant associations across outcomes, indicating that different dimensions of empowerment may have varying influences on fertility preferences and behaviours.

Previous studies have also highlighted the differentiated effects of women’s empowerment on reproductive health outcomes [12, 38]. The inconclusive correlations between empowerment indicators and outcomes, as noted in other research [52, 53], reflect the inherent multidimensionality and complexity of the empowerment construct. This complexity poses challenges to establish standardized and universally valid measurements [15, 20, 54, 55]. While empowerment indicators may not precisely capture changes in women’s capacity to make choices, they remain valuable in illustrating the broader context and pathways of change [13].

### Participation in decision-making

This study found that women’s participation in household decision-making was statistically significant and positively associated with achieving fertility preferences–that is, alignment between number of children ever born and the ideal number of children. However, this empowerment indicator showed no significant association with either the total number of children ever born and the ideal number of children.

These findings highlight the complex and multidimensional nature of women’s empowerment in the Indonesian context. It is possible that while decision-making power enhances a woman’s ability to realize her fertility preferences within her personal and social circumstances, broader cultural, religious or familial norms may still heavily influence the actual number of children a woman has or desires. Fertility-related decisions are often negotiated within households and shaped by community expectations, which may dilute the direct influence of individual decision-making autonomy on fertility behaviours. However, greater involvement in household decisions may increase a woman’s ability to realize her own preferences within those constraints.

Previous research has demonstrated inconsistent associations between decision-making and fertility outcomes. For example, studies in Pakistan [33], Mozambique [26], Burkina Faso, Mali, Niger and Chad [15] reported that disempowered women tend to have more children and a higher ideal number of children than empowered women. In contrast, other studies have shown that joint decision-making contributes to lower rates of domestic violence and higher contraceptive use [31, 44, 56–60]. Within Indonesia, some evidence suggests that empowered women tend to express lower ideal fertility and have reduced unmet need for family planning [12, 61]. The mixed findings across contexts and outcomes underscore the need to interpret empowerment as a context-specific and domain-specific construct, rather than a uniform measure with universally consistent effects.

### Attitude towards wife beating

Rejecting all justifications for wife beating was the only empowerment indicator significantly associated with all three fertility outcomes: total number of children ever born, ideal number of children, and fertility preference. This suggests that women who do not accept violence as normative may be more empowered in asserting their reproductive choices and controlling their fertility trajectories.

This finding aligns with previous research in Indonesia, where acceptance of wife beating has been associated with higher levels of unmet family planning needs and reduced autonomy in reproductive decision-making [61]. The link between attitudes toward domestic violence and fertility behaviour may reflect broader dimensions of gender inequality and power imbalances within households. In contexts where violence is normalized, women may have limited ability to negotiate contraceptive use or express preferences about childbearing.

International studies support this interpretation. For example, research in Bangladesh and the Philippines found that women with lower empowerment levels–often reflected in tolerating domestic or spousal violence–were more likely to have a higher number of living children and express a higher ideal number of children [34, 62]. Similarly, studies from Guinea, Zambia, and Mali reported that women with more negative attitudes toward wife beating tended to report lower ideal family sizes [53]. Together, these findings underscore the importance of addressing gender-based violence and its normalization as a critical factor in empowering women and shaping fertility-related outcomes.

### Attitude towards refusing sex

Women’s attitude towards refusing sex with their husbands was positively associated with the number of children ever born and the ideal number of children. This aligns with findings from Mozambique, Timor Leste, Guinea, and Zambia [24–27], where greater control over sexual relations was linked to improved reproductive autonomy and fertility planning. In the Indonesian context, cultural and gender norms often position women in less empowered roles regarding sexual decision-making. A prior study in Indonesia, as well as a pooled analysis across 31 sub-Saharan African countries, suggested that participation in household decision-making strengthens women’s bargaining power within marital sexual relationships [30, 45]. Our findings further support the notion that woman’s perceived right to refuse sex may influence fertility intentions and outcomes through its connection with broader empowerment processes.

### Labour force participation

The labour force participation indicator showed a somewhat counterintuitive association: women who were employed were more likely to have a higher number of children compared to those not engaged in paid work. This aligns with findings from a previous Indonesian study [12], which also reported a significant association between women’s employment and a higher ideal number of children.

The inconclusive nature of this relationship suggests that labour force participation alone may be an insufficient proxy for empowerment. A more nuanced understanding would require incorporating additional variables, such as decision-making power over earnings, ownership of assets (e.g., bank accounts, land, or housing), the type and stability of employment, and relative income compared to their spouse [8, 35, 49, 63, 64]. These dimensions could offer a more comprehensive assessment of how economic participation intersects with reproductive decision-making.

### Women’s empowerment index

In this study, the level of women’s empowerment was measured using 11 indicator questions as part of the descriptive statistics. While several previous studies have included contraceptive use as either an indicator of empowerment or a fertility-related outcome [31, 32, 36, 43, 44, 65], we chose not to include this variable. This decision was based on the relatively high prevalence of modern contraceptive use in Indonesia [6, 66], which limits its discriminatory power in this context. Other frequently cited indicators of women’s autonomy, such as media exposure [41, 55, 67] and community participation [64], were also excluded. The majority of women in our sample already reported media exposure (newspaper, radio, and television), reducing the usefulness of this variable in distinguishing levels of empowerment. Additionally, the IDHS dataset did not include important dimensions such as participation in public life, power relations beyond the marital context, or women’s engagement in political and social leadership roles [68], which are critical for a more comprehensive assessment of empowerment.

### Strengths and limitations

To our knowledge, this is the first study to examine three different fertility-related outcomes– total number of children ever born, ideal number of children, and fertility preference–using nationally representative data from the 2017 IDHS. By applying an outcome-wide analytical approach, this study provides a more comprehensive understanding of how different dimensions of women’s empowerment are associated with reproductive behaviour and preferences. Additionally, this study employed PRs rather than ORs to estimate associations with the binary outcome, thereby reducing the risk of overestimation, especially in cases where the outcome is common.

This study has several limitations. First, key sociodemographic factors such as religion and ethnicity, which are known to influence fertility behaviour and gender-related norms [11, 14, 41, 69], were not included as covariates, as these variables were not available in the 2017 IDHS dataset. Similarly, regional variations were not analysed by province, despite known geographic differences in empowerment and fertility behaviours across Indonesia’s 34 provinces. Local traditions, beliefs, and sociocultural contexts may account for regional disparities that were not captured in this analysis.

Second, both the women’s empowerment index and the three fertility outcomes reflect a static snapshot as of 2017. While this provides a valid cross-sectional view of differences in fertility preferences and behaviours between empowered and less empowered women, the dynamic nature of empowerment and reproductive decision-making over time cannot be captured. Additionally, the empowerment index used in this study reflects national averages and does not fully account for sub-national disparities or more nuanced dimensions of empowerment, such as community participation, engagement in political life, or decision-making beyond the household. One important limitation of this study relates to the measurement of fertility preference. Our operationalization compares the number of children ever born with the ideal number of children reported by women of reproductive age (15–49 years). However, many women in this age range may not yet have completed their childbearing, which introduces uncertainty regarding whether their stated preferences will ultimately be fulfilled. As such, our measure of fertility preference may overestimate the extent to which women’s reproductive goals have not been met, particularly among younger respondents. Due to limitations in sample size, we were unable to restrict this analysis to only women with completed fertility. This limitation should be considered when interpreting the findings.

Third, the use of cross-sectional data limits the ability to draw causal inferences between empowerment and fertility outcomes. Future research would benefit from longitudinal designs that could better capture the temporal relationship between changes in empowerment and reproductive behaviour [19, 51, 53, 70].

### Policy implication

This study found that, on average, women’s ideal number of children exceeded with the number of children ever born, indicating a gap between fertility aspirations and outcomes. Although Indonesia’s TFR was relatively low TFR 2.18 in 2020 [2], it remained slightly above the Replacement Level Fertility of 2.1 needed for stable population growth [43, 44]. According to the National Population and Family Planning Board (BKKBN), the national birth control program remains on track [71, 72]. However, significant disparities in fertility persist across provinces–for example, TFR in Jakarta is as low as 1.75, while East Nusa Tenggara was 2.79 [2, 73]. These regional differences call for more targeted family planning policies and localized approaches to address demographic imbalances.

Only 19% of women in this study population achieved the highest score on the empowerment index, underscoring the urgency for policymakers to prioritize gender equality and women’s empowerment to achieve SDG 5. The data on women’s empowerment were drawn from the 2017 IDHS, which may not fully reflect current levels of empowerment or recent social and policy changes. Although the 2024 IDHS is currently underway, updated results were not available at the time of writing. Nonetheless, structural barriers to gender equality remain, and the exclusion of gender-based violence from Indonesia’s SDG 5 monitoring framework indicates persistent policy and data gaps. These findings underscore the continued relevance of prioritizing gender-sensitive policies and sustained efforts to advance women’s empowerment.

The more recent evaluations from 2022 to 2023 showed that Indonesia is still categorized as “moderately improving” on gender equality, with only four indicators being reported: demand for family planning satisfied by modern methods, female education, female labour force participation, and the proportion of seats held by women in the national parliament [46, 74, 75]. Notably, indicators related to gender-based violence (GBV) are not currently included in Indonesia’s SDG reporting framework, suggesting a major gap in the national monitoring of progress toward gender equity. Addressing this omission and enhancing the empowerment of women—particularly through expanded data collection, legal reform, and inclusive policy design—will be crucial for advancing reproductive autonomy and sustainable development.

## Conclusions

This study highlights the complex and multidimensional nature of women’s empowerment and its varied associations with fertility outcomes and preferences among married women in Indonesia. The findings emphasize the value of a domain-specific approach to better understand and measure empowerment in relation to reproductive health. Notably, indicators related to attitudes toward gender-based violence emerged as particularly influential, suggesting that addressing domestic violence is critical to supporting women’s reproductive autonomy.

Given that gender-based violence indicators are currently not included in Indonesia’s SDG 5 monitoring framework, this study calls attention to an important gap in national efforts toward achieving gender equality. Strengthening data systems and policies to better reflect and address these dimensions of empowerment is essential. Continued investment in women’s empowerment remains vital to ensure informed and autonomous reproductive choices in the context of Indonesia’s demographic transition.

## Electronic supplementary material

Below is the link to the electronic supplementary material.

Supplementary Material 1

## Data Availability

The data used in this study are from the DHS program, which are publicly available. The DHS data can be accessed through the DHS program website (https://dhsprogram.com) upon registration and approval. The specific datasets used in this study include the Individual Women’s Data - Individual Recode (IR) from the 2017 Indonesia DHS. Researchers can request access to these datasets by following the instructions provided on the DHS Program website.

## Abbreviations

BKKBN: The National Population and Family Planning Board
DHS: Demographic and Health Survey
GBV: Gender-Based Violence
IDHS: Indonesia Demographic and Health and Survey
OR: Odds Ratio
PR: Prevalence Ratio
SDGs: Sustainable Development Goals
TFR: Total Fertility Rate
