# Supplementary material for: The Role of Women’s Empowerment in Fertility Preferences and Outcomes: Analysis of the 2017 Indonesia Demographic and Health Survey
Source: BMC Womens Health. 2025 Apr 30;25:211. doi: 10.1186/s12905-025-03748-6 (PMC12042363; doi:10.1186/s12905-025-03748-6)
Supplement: Supplementary file 1 — Supplementary Material 1 [file 12905_2025_3748_MOESM1_ESM.docx]

**Appendix**

**Table 1. Poisson Regression Output: Outcome Total Number of Children Ever Born (Weighted 2017 IDHS Data)**

| **Variables** | **Labels** | **Simple Poisson Regression** | | | **Model 1**  **(adjusted for 4 women empowerment variables)** | | | **Model 2**  **(adjusted for covariates)** | | | **Model 3**  **(full model)** | | |
| --- | --- | --- | --- | --- | --- | --- | --- | --- | --- | --- | --- | --- | --- |
|  |  | β | 95% CI | p-val | β | 95% CI | p-val | β | 95% CI | p-val | β | 95% CI | p-val |
| **Participation in decision-making** | Yes  No | Ref  0.0105 | –0.0074 to 0.0284 | 0.25 | Ref  0.0032 | –0.0147 to 0.0210 | 0.73 |  | | | Ref  0.0075 | –0.0089 to 0.0239 | 0.37 |
| **Disagreeing with reasons justifying wife beating** | Yes  No | Ref  0.0256 | 0.0078 to 0.0434 | <0.01 | Ref  0.0219 | 0.0043 to 0.0395 | <0.05 |  |  |  | Ref  0.0292 | 0.0136 to 0.0447 | <0.001 |
| Attitude towards refusing sex with husband | Yes  No | Ref  0.1304 | 0.1136 to 0.1473 | <0.001 | Ref  0.1292 | 0.1123 to 0.1461 | <0.001 |  |  |  | Ref  0.0231 | 0.0078 to 0.0384 | <0.01 |
| **Labor force participation** | Yes  No | Ref  –0.0325 | –0.0499 to –0.0150 | <0.001 | Ref  –0.0302 | –0.0475 to –0.0128 | <0.001 |  |  |  | Ref  0.0361 | 0.0206 to 0.0515 | <0.001 |
| **Age groups** | 22–29  30–39  40–49 | Ref  0.5476  0.7788 | 0.5297 to 0.5654  0.7579 to 0.7997 | <0.001  <0.001 |  | | | Ref  0.5357  0.7458 | 0.5179 to 0.5535  0.7248 to 0.7669 | <0.001  <0.001 | Ref  0.5404  0.7540 | 0.5223 to 0.5584  0.7325 to 0.7754 | <0.001  <0.001 |
| **Education wife** | Higher  Secondary  Primary  No Edu | Ref  0.1858  0.4260  0.6748 | 0.1604 to 0.2111  0.3975 to 0.4543  0.6090 to 0.7405 | <0.001  <0.001  <0.001 |  |  |  | Ref  0.1693  0.2507  0.3550 | 0.1439 to 0.1946  0.2194 to 0.2820  0.2895 to 0.4206 | <0.001  <0.001  <0.001 | Ref  0.1577  0.2357  0.3385 | 0.1320 to 0.1833  0.2039 to 0.2674  0.2726 to 0.4039 | <0.001  <0.001  <0.001 |
| **Education husband** | Higher  Secondary  Primary  No Edu | Ref  0.0913  0.2767  0.4914 | 0.0646 to 0.1180  0.2476 to 0.3058  0.4227 to 0.5602 | <0.001  <0.001  <0.001 |  |  |  | Ref  –0.0132  –0.0090  0.0703 | –0.0393 to 0.0129  –0.0404 to 0.0223  0.0048 to 0.1358 | 0.32  0.57  <0.05 | Ref  –0.0127  –0.0080  0.0697 | –0.0387 to 0.0134  –0.0393 to 0.0232  0.0040 to 0.1354 | 0.34  0.61  <0.05 |
| **Wealth quintile** | Richest  Richer  Middle  Poorer  Poorest | Ref  –0.0032  0.0263  0.0929  0.2495 | –0.0262 to 0.0197  0.0020 to 0.0506  0.0672 to 0.1182  0.2185 to 0.2806 | 0.78  <0.005  <0.001  <0.001 |  |  |  | Ref  –0.0185  0.0005  0.0545  0.1894 | –0.0409 to 0.0040  –0.0252 to 0.0242  0.0273 to 0.0817  0.1549 to 0.2238 | 0.11  0.97  <0.001  <0.001 | Ref  –0.0188  0.0028  0.0506  0.1845 | –0.0411 to 0.0036  –0.0273 to 0.0218  0.0234 to 0.0778  0.1503 to 0.2187 | 0.09  0.83  <0.001  <0.001 |
| **Place of residence** | Urban  Rural | Ref  0.0783 | 0.0584 to 0.0983 | <0.001 |  |  |  | 0.0024 | –0.0173 to 0.0222 | 0.81 | Ref  0.0010 | –0.0186 to 0.0206 | 0.92 |

**Table 2. Poisson Regression Output: Outcome Perception of Ideal Number of Children (Weighted 2017 IDHS Data)**

| **Variables** | **Labels** | **Simple Poisson Regression** | | | **Model 1**  **(adjusted for 4 women empowerment variables)** | | | **Model 2**  **(adjusted for covariates)** | | | **Model 3**  **(full model)** | | |
| --- | --- | --- | --- | --- | --- | --- | --- | --- | --- | --- | --- | --- | --- |
|  |  | β | 95% CI | p-val | β | 95% CI | p-val | β | 95% CI | p-val | β | 95% CI | p-val |
| **Participation in decision-making** | Yes  No | Ref  0.0223 | 0.0059 to 0.0387 | <0.01 | Ref  0.0120 | –0.0044 to 0.0283 | 0.15 |  | | | Ref  0.0117 | –0.0042 to 0.0276 | 0.15 |
| **Disagreeing with reasons justifying wife beating** | Yes  No | Ref  0.0468 | 0.0320 to 0.0615 | <0.001 | Ref  0.0436 | 0.0290 to 0.0581 | <0.001 |  |  |  | Ref  0.0400 | 0.0256 to 0.0544 | <0.001 |
| Attitude towards refusing sex with husband | Yes  No | Ref  0.1134 | 0.0973 to 0.1295 | <0.001 | Ref  0.1121 | 0.0961 to 0.1281 | <0.001 |  |  |  | Ref  0.0727 | 0.0568 to 0.0886 | <0.001 |
| **Labor force participation** | Yes  No | Ref  0.0023 | –0.0118 to 0.0165 | 0.75 | Ref  0.0044 | –0.0095 to 0.0182 | 0.54 |  |  |  | Ref  0.0201 | 0.0060 to 0.0342 | <0.01 |
| **Age groups** | 22–29  30–39  40–49 | Ref  0.0587  0.1279 | 0.0438 to 0.0735  0.1109 to 0.1448 | <0.001  <0.001 |  | | | Ref  0.0534  0.1109 | 0.0384 to 0.0684  0.0931 to 0.1288 | <0.001  <0.001 | Ref  0.0553  0.1129 | 0.0402 to 0.0705  0.0948 to 0.1309 | <0.001  <0.001 |
| **Education wife** | Higher  Secondary  Primary  No Edu | Ref  –0.0130  0.1031  0.2921 | –0.0317 to 0.0057  0.0799 to 0.1263  0.2388 to 0.3454 | 0.17  <0.001  <0.001 |  |  |  | Ref  0.0011  0.0688  0.2010 | –0.0214 to 0.0236  0.0400 to 0.0975  0.1437 to 0.2582 | 0.92  <0.001  <0.001 | Ref  –0.0087  0.0503  0.1713 | –0.0314 to 0.0139  0.0216 to 0.0789  0.1148 to 0.2278 | 0.45  <0.001  <0.001 |
| **Education husband** | Higher  Secondary  Primary  No Edu | Ref  –0.0423  0.0463  0.1575 | –0.0623 to –0.0223  0.0221 to 0.0704  0.1020 to 0.2129 | <0.001  <0.001  <0.001 |  |  |  | Ref  –0.0835  –0.0801  –0.0511 | –0.1075 to –0.0594  –0.1097 to –0.0506  –0.1069 to 0.0047 | <0.001  <0.001  0.07 | Ref  –0.0833  –0.0813  –0.0565 | –0.1070 to 0.0595  –0.1104 to 0.0519  –0.1130 to -0.0001 | <0.001  <0.001  <0.05 |
| **Wealth quintile** | Richest  Richer  Middle  Poorer  Poorest | Ref  0.0106  0.0428  0.0577  0.1748 | –0.0107 to 0.0320  0.0192 to 0.0663  0.0329 to 0.0826  0.1460 to 0.2035 | 0.33  <0.001  <0.001  <0.001 |  |  |  | Ref  0.0237  0.0499  0.0540  0.1522 | 0.0014 to 0.0461  0.0247 to 0.0752  0.0251 to 0.0828  0.1183 to 0.1862 | <0 05  <0.001  <0.001  <0.001 | Ref  0.0217  0.0445  0.0468  0.1379 | –0.0008 to 0.0441  0.0192 to 0.0697  0.0179 to 0.0758  0.1041 to 0.1716 | 0.06  <0.001  <0.01  <0.001 |
| **Place of residence** | Urban  Rural | Ref  0.0645 | 0.0444 to 0.0845 | <0.001 |  |  |  | Ref  0.0153 | –0.0065 to 0.0371 | 0.17 | Ref  0.0111 | –0.0105 to 0.0327 | 0.31 |

**Table 3. Poisson Regression Output: Prevalence Ratio for Outcome Fertility Preference (Weighted 2017 IDHS Data)**

| **Variables** | **Labels** | **Simple Poisson Regression** | | | **Model 1**  **(adjusted for 4 women empowerment variables)** | | | **Model 2**  **(adjusted for covariates)** | | | **Model 3**  **(full model)** | | |
| --- | --- | --- | --- | --- | --- | --- | --- | --- | --- | --- | --- | --- | --- |
|  |  | PR | 95% CI | p-val | PR | 95% CI | p-val | PR | 95% CI | p-val | PR | 95% CI | p-val |
| **Participation in decision-making** | Yes  No | –  1.04 | 1.02 – 1.06 | <0.001 | –  1.03 | 1.01 – 1.05 | <0.01 |  | | | 1.01 | 1.01 – 1.02 | <0.01 |
| **Disagreeing with reasons justifying wife beating** | Yes  No | –  1.06 | 1.04 – 1.08 | <0.001 | –  1.05 | 1.04 – 1.07 | <0.001 |  |  |  | 1.02 | 1.01 – 1.02 | <0.001 |
| Attitude towards refusing sex with husband | Yes  No | –  1.00 | 0.98 – 1.02 | 0.75 | –  1.00 | 0.98 – 1.02 | 0.79 |  |  |  | 1.01 | 0.99 – 1.01 | 0.08 |
| **Labor force participation** | Yes  No | –  1.03 | 1.01 – 1.05 | <0.01 | –  1.03 | 1.01 – 1.05 | <0.01 |  |  |  | 1.00 | 0.99 – 1.01 | 0.92 |
| **Age groups** | 22–29  30–39  40–49 | –  0.77  0.75 | 0.76 – 0.79  0.73 – 0.77 | <0.001  <0.001 |  | | | 0.90  0.89 | 0.89 – 0.91  0.88 – 0.90 | <0.001  <0.001 | 0.90  0.89 | 0.89 – 0.91  0.88 – 0.90 | <0.001  <0.001 |
| **Education wife** | Higher  Secondary  Primary  No Edu | –  0.93  0.88  0.93 | 0.90 – 0.95  0.86 – 0.91  0.87 – 0.99 | <0.001  <0.001  <0.05 |  |  |  | 0.97  0.97  0.99 | 0.96 – 0.98  0.95 – 0.98  0.96 – 1.02 | <0.001  <0.001  0.60 | 0.97  0.96  0.98 | 0.96 – 0.98  0.95 – 0.98  0.96 – 1.01 | <0.001  <0.001  0.35 |
| **Education husband** | Higher  Secondary  Primary  No Edu | –  0.94  0.90  0.92 | 0.92 – 0.96  0.88 – 0.93  0.86 – 0.99 | <0.001  <0.001  <0.05 |  |  |  | 0.97  0.97  0.97 | 0.96 – 0.99  0.95 – 0.98  0.94 – 1.01 | <0.001  <0.001  0.11 | 0.98  0.97  0.97 | 0.96 – 0.99  0.95 – 0.98  0.94 – 1.00 | <0.001  <0.001  0.10 |
| **Wealth quintile** | Richest  Richer  Middle  Poorer  Poorest | –  1.02  1.05  1.03  1.08 | 0.99 – 1.05  1.02 – 1.08  1.01 – 1.06  1.05 – 1.12 | 0.16  <0.01  <0.05  <0.001 |  |  |  | 1.02  1.03  1.03  1.05 | 1.01 – 1.03  1.02 – 1.05  1.02 – 1.04  1.04 – 1.07 | <0.01  <0.001  <0.001  <0.001 | 1.02  1.03  1.03  1.05 | 1.01 – 1.03  1.02 – 1.05  1.01 – 1.04  1.04 – 1.07 | <0.01  <0.001  <0.001  <0.001 |
| **Place of residence** | Urban  Rural | –  1.00 | 0.98 – 1.02 | 0.81 |  |  |  | 0.99 | 0.98 – 0.99 | <0.05 | 0.99 | 0.98 – 0.99 | <0.01 |
